# An explanatory mixed methods study assessing Canadian chiropractors’ attitudes and orientation toward patient-centred care

**DOI:** 10.1186/s12998-025-00610-2

**Published:** 2025-10-22

**Authors:** Kent Jason Stuber, Silvano Mior, Gina Dolan, Mark Langweiler, Peter W. McCarthy

**Affiliations:** 1 Parker University Research Center, Dallas, USA; 2 Canadian Memorial Chiropractic College, Toronto, Canada; 3 Institute for Disability and Rehabilitation Research at CMCC and Ontario Tech University, Oshawa, Canada; 4 Faculty of Life Sciences and Education, University of South Wales, Pontypridd, UK; 5 London South Bank University, London, UK; 6 University of South Wales, Pontypridd, UK; 7 Faculty of Health Sciences, Durban University of Technology, Durban, South Africa

**Keywords:** Chiropractic, Patient-centredness, Mixed methods, Patient-practitioner orientation scale, Attitudes

## Abstract

**Background:**

Patient-centred care (PCC) is considered an essential concept in twentyfirst-century healthcare; however, health care providers’ attitudes can be an important barrier or facilitator to its actual implementation. Chiropractic is frequently referred to as providing PCC. However, the attitudes of practising chiropractors towards patient-centred care have not previously been evaluated.

**Objectives:**

To explore attitudes and orientation of chiropractors towards patient-centered care.

**Methods:**

We used a sequential explanatory mixed methods with quantitative priority study design. Data were collected from May 2018 to April 2019 from a convenience sample of Canadian chiropractors located across Canada. Quantitative data were collected by a survey consisting of demographic and clinical practice questions, and the Patient-Practitioner Orientation Scale (PPOS) to measure practitioner attitudes towards care delivered (range 1–6, scores < 4.57 = doctor-centred attitudes). Qualitative data were collected using semi-structured interviews. Quantitative data were analyzed with descriptive statistics and bivariate analysis; whilst qualitative data were analyzed using thematic analysis.

**Results:**

We included 31 chiropractors in the study, with seven completing interviews. The mean for PPOS Overall score was 4.35 (95% CI [4.18, 4.52]) and was found to be significantly associated with years in practice and number of patients seen per week. The average PPOS Sharing and PPOS Caring scores were 4.20 (95% CI 3.95–4.45) and 4.50 (95% CI 4.35–4.66), respectively. Number of patients seen per week and per hour were significant predictors of the PPOS Sharing score; whilst years in practice was a significant predictor of PPOS Caring and Overall scores. Interview data supported the association between greater exposure to patients and more patient-centred attitudes among chiropractors.

**Conclusions:**

In our sample of Canadian chiropractors, doctor-centred attitudes towards care prevailed, comparable to that reported for other health professions. The significant association between patient-centred attitudes and patient load and clinical experience suggests that chiropractors may learn such attitudes through experience. Future research to further explore chiropractors’ attitudes and orientation towards care using the PPOS or a similar instrument are required.

**Supplementary Information:**

The online version contains supplementary material available at 10.1186/s12998-025-00610-2.

## Background

Patient-centred care (PCC) has emerged as an essential concept in twentyfirst-century healthcare. Patient-centred care was defined by the Institute of Medicine in 2001 as “care that is respectful of and responsive to individual patient preferences, needs, and values ensuring that patient values guide all clinical decisions (p. 40)” [1]. PCC represents a core value of practice and a paradigm shift that challenges traditional healthcare models by prioritizing the partnership between healthcare professionals (HCPs) and patients in decision-making and care planning [2–5]. In a PCC model, HCPs have a comprehensive understanding of the patient and consider patient’s needs, preferences, and values [6–11]. Previous research has demonstrated that PCC can positively impact patient satisfaction with their care, involvement in the decisions made around their care, and potentially clinical outcomes and costs [12–16].

While there is increasing recognition and emphasis on PCC, there remain implementation challenges, including the roles and attitudes of HCPs [17–20]. HCPs represent the faces of healthcare during patient visits, and their attitudes can be an important barrier or facilitator to the actual provision of PCC. HCP background and training, knowledge of or skills in PCC, clinical experience, professional culture, and organizational factors can all influence their attitudes toward and ability to practice PCC [18–22]. To date, it is uncertain to what extent HCP attitudes impact clinical behaviours, communication patterns, and the ability to forge a trusting provider-patient relationship. Thus, research focussed on understanding such attitudes can aid with its further integration into practice.

Despite members of the chiropractic profession describing their approach to patient care as involving wholly or elements of PCC [23, 24] there is little research supporting or refuting such an assertion. Therefore, an initial step in assessing the extent to which chiropractors are patient-centred is to determine their attitudes toward PCC. Our study aimed to explore the orientation toward patient-centred care among chiropractors participating in a practice-based study.

## Methods

A sequential explanatory mixed methods study with a quantitative priority was used [25]. This design facilitates the explanation and contextualization of the quantitative findings [25]. The study received ethics approval from the University of South Wales Schools of Health, Sport and Professional Practice and Care Sciences Research Ethics SubGroup (2017KS1101) and the Canadian Memorial Chiropractic College’s Research Ethics Board (Project 172027, #1712X01). All participants provided informed consent prior to participation in this study. Data was collected from May 2018 to April 2019.

### Settings and inclusion criteria

We used a convenience sample to purposefully recruit chiropractors who practiced in clinics that were participating in a practice-based study involving chiropractic patients [26, 27]. Using a purposeful sampling strategy, we attempted to include participants with diverse personal and practice characteristics. Thus, we aimed to recruit clinics of varying sizes, locations (rural, suburban, and urban), and practice models (multidisciplinary, solo practice, etc.) in our sample. We also included male and female chiropractors with varying years of clinical experience. These clinics were located in one of seven Canadian provinces (3 in British Columbia, 3 in Alberta, 3 in Saskatchewan, 1 in Manitoba, 6 in Ontario, 2 in Nova Scotia, and 2 in Newfoundland). The clinics and chiropractors involved in this study were recruited based on professional networks and their willingness to participate At least one of the chiropractors in most of the clinics (n = 16) were known to the primary author (KS) through professional and research contacts.

### Quantitative component

#### Questionnaire

A brief questionnaire was developed that included demographic information (gender, number of years in practice, the chiropractic educational institution where they trained), practice information (number of patients seen per week, hours per week spent seeing patients, and the style of their practice, i.e., whether multidisciplinary, solo practice, etc.), and the Patient-Practitioner Orientation Scale (PPOS) [28]. The questionnaire was assessed for face validity by the study team and a practicing chiropractor, and as a consequence of this review, no additional changes were made.

The PPOS is a self-report survey completed by patients or practitioners to assess attitudes toward the relationship between practitioners and their patients, including the sharing of power and control between them [29]. The PPOS consists of 18 multiple-choice items, each on a 6-point scale with detractors ranging from “Strongly Disagree” which is scored as 1 on most items to “Strongly Agree” which is scored as 6 on most items. The PPOS provides an Overall score and scores on two subscales, Sharing and Caring. The Sharing subscale items assess attitudes towards sharing information, power, control, and decision-making between practitioners and patients. The Caring subscale items assess attitudes towards warm doctor-patient relationships, and the consideration of psychosocial factors and a holistic approach to care. The Overall PPOS and subscale scores each range between 1 and 6, where higher scores indicate more patient-centred attitudes, while lower scores are indicative of more disease-centred or doctor-centred attitudes. Krupat et al. [28] defined mean PPOS scores as “doctor-centred” if they fell below the cutoff score of 4.57, whereas scores of 5.00 or greater were considered “patient-centred”, while scores between 4.57 and 5.00 were categorized as “medium”.

The PPOS is a validated tool that was selected as it has been used in studies involving different health professions including medicine and nursing [30–35], and student populations [36], including those in medicine [9, 10, 13–20], chiropractic [37], physiotherapy [38], orthodontics [39], and multiple health disciplines [33, 40]. The PPOS has been translated into several languages and used in numerous countries [31, 41–49]. The construct validity of the PPOS has also been demonstrated [50].

#### Study flow

We distributed five study packages to each of the 20 involved clinics. Each study package consisted of a participant information sheet, informed consent form, and the questionnaire. The chiropractors completed the informed consent form and the questionnaire, which included the demographic and practice information and the PPOS. The completed informed consent and questionnaire from the chiropractor were each placed in a separate envelope, sealed and returned to the primary author by secure courier.

#### Data analysis

Questionnaire data were entered into a password protected Microsoft Excel™ spreadsheet and imported into SPSS Version 25 (IBM Corp. Released 2017. IBM SPSS Statistics for Windows, Version 25.0. Armonk, NY: IBM Corp) for analysis. These data were descriptively analyzed, calculating means with standard deviations (SD), medians, ranges for continuous independent variables, and counts and percentages for categorical independent variables.

We used the PPOS Overall and the Sharing and Caring subscale scores as dependent variables for determining chiropractor patient-centred communication and attitudes. The PPOS Overall and subscale scores are each continuous variables for which means, ranges, and 95% Confidence Intervals (CIs) were calculated.

The association between independent variables selected from the chiropractor demographic and practice information questions and the dependent variables was assessed using bivariate analysis. PPOS score means of the categorical independent variables were compared using the independent samples t-test and one-way ANOVA. Correlations between continuous variables were determined using Pearson’s test. Correlation coefficients up to 0.3 were considered weak, from 0.4 to 0.6 were considered moderate, and those above 0.7 were considered strong [51].

#### Qualitative component

A representative sample of chiropractors was purposefully recruited from those who completed the study questionnaire. This sample included male and female chiropractors, of varying amounts of clinic experience, and from different Canadian provinces. One of the authors (KS), who has formal training and experience with qualitative research, conducted the individual semi-structured interviews. The interviewer was a practicing chiropractor with nearly 20 years of clinical experience. The interview guide was developed to explain the quantitative findings and further explore chiropractor’s experiences and perspectives on providing PCC to patients with chronic musculoskeletal conditions. The interview guide was informed by Mead and Bower’s conceptual framework of patient-centredness [7], the Chronic Care Model [52], information from a pilot study [53], and the study’s questionnaire results (see Appendix 1). The interviews were conducted in-person when possible, otherwise by telephone or videoconference. The interviews were audio recorded, transcribed verbatim by a professional transcriptionist, and a random selection of 25% were checked for transcription accuracy.

Qualitative data analysis was conducted inductively using thematic analysis as described by Braun and Clarke, involving six phases [54]. The first phase involved *familiarization and transcription of data*. Two authors (KS and GD) independently analyzed each transcript following multiple readings. The second phase involved *generating initial codes*, with the same two authors independently coding all of the transcripts, generating codes de novo. The authors then met to discuss and reach consensus on coding, and a third author (SM) resolved disagreements when necessary. In the third phase, the same two authors *searched for themes* from the generated codes and qualitative data. The fourth phase involved the same authors independently *reviewing themes* from the initially generated themes and subthemes, again meeting to discuss and refine emerging themes. The fifth phase involved formally *defining and naming themes*, where the entire study team confirmed the identified themes and subthemes, along with suitable data extracts (quotes). The sixth and final phase involved *producing the report*; the final themes and their definitions will appear in a subsequent manuscript.

Qualitative data collection and analysis continued until data saturation was achieved, and the authors agreed that no new codes emerged, ceasing data collection and theme development. Bracketing was employed as the interviewer is a chiropractor and had previous contact and professional interactions with the interview participants [55]. Bracketing is an approach used in qualitative research whereby the researchers intentionally set aside their preconceptions, biases, and prior knowledge about the topic under study to enhance the rigor and credibility of data collection and analysis [55]. The Primary Investigator (KS) also maintained a reflective journal that was logged throughout the research process to identify and limit any preconceptions of the topic and potential bias [55].

#### Reporting and integration

The study methods and results were reported in adherence to the Good Reporting of A Mixed Methods Study (GRAMMS, see Appendix 2) [56]. The quantitative and qualitative data were integrated throughout the study from the design, methods, interpretation, and reporting stages [57]. The quantitative and qualitative samples were connected as the chiropractors that completed the interview component previously completed the questionnaire [57]. The questionnaire results were considered when developing the interview schedules for the qualitative interviews [25, 58, 59]. Qualitative findings were used to potentially explain the results of the overall PPOS scores, providing examples of quotes representative of patient-centred or doctor-centred attitudes where applicable [59].

## Results

A total of 31 completed questionnaires from the 100 distributed were included in the analysis. Three additional questionnaires were returned but excluded from analysis: one due to an incomplete informed consent form and two because the demographic and practice pattern questions were not filled out. The remaining 66 questionnaires were not returned.

### Chiropractor demographic characteristics and practice patterns

The demographic and practice characteristics of the participating chiropractors are presented in Table 1. Most chiropractors were males (67.7%), received their chiropractic training in Canada (80.6%), worked in a multidisciplinary setting (80.6%), with an average of about 15 years of practice experience. On average, they spent 31.85 h per week in patient care, saw about 88 patient visits per week, or 3 patients per hour. From this sample of chiropractors, seven completed the individual semi-structured interviews, including two females and five males, six of whom graduated from Canadian chiropractic institutions, and one from an American program. These chiropractors had been in practice for a mean of 22.3 years (range 14 to 40 years), and practised in four Canadian provinces (British Columbia, Alberta, Saskatchewan, and Ontario). This sample was younger and did not include a chiropractor from Atlantic Canada compared to the overall participating chiropractors. The interviews lasted 46.3 min on average, ranging between 32 and 73 min.

**Table 1:** Chiropractor demographic and practice data (n = 31)

Item	Result
Gender	21 male
	10 female
Years in practice	
Mean (SD)	15.08 (12.41)
Median	16
Range	1–45
Country where received chiropractic education	25 Canada
	6 United States
Province of practice	13 Ontario
	6 Saskatchewan
	4 Alberta
	3 British Columbia
	2 Manitoba
	2 Nova Scotia
	1 Newfoundland
Practice setting	25 Multidisciplinary
	6 Multiple chiropractors
Hours seeing patients per week	
Mean (SD)	31.85 (10.09)
Median	30
Range	13–55
Patients seen per week	
Mean (SD)	88.45 (55.44)
Median	80
Range	15–250
Patients seen per hour	
Mean (SD)	2.80 (1.54)
Median	2.86
Range	0.68–6.94

SD, Standard deviation

### PPOS results

Table 2 presents the PPOS scores reported by participating chiropractors. PPOS Overall and subscale scores were assessed for normal distribution using the Shapiro–Wilk and Kolmogorov–Smirnov tests and found to be normally distributed [60, 61]. The average PPOS Overall score was 4.35, while the average score on the PPOS Sharing subscale was 4.20 and 4.50 on the Caring subscale.

**Table 2:** Chiropractor PPOS scores (n = 31)

	PPOS overall	PPOS sharing	PPOS caring
Mean (95% CI)	4.35 (4.18–4.52)	4.20 (3.95–4.45)	4.50 (4.35–4.66)
Range	3.61–5.44	3.11–5.56	3.78–5.67

PPOS, Patient practitioner orientation scale; CI, Confidence interval

The internal consistency of the PPOS is moderate (0.73 for the PPOS Total score, 0.67 for the Sharing subscale and 0.52 for the Caring subscale) [28]. The higher PPOS Caring subscale scores could potentially be attributed to chiropractors focusing not just on physical symptoms or conditions but on a patient’s entire life holistically, particularly for patients with chronic conditions:

“*Chronic conditions involve a lot more beliefs, attitudes, prior history, and habits that need to be looked at versus just a very simple kind of new (acute) problem…. It’s not just about the physical movements or pain; it’s about their whole life*” (DC5).”

Another chiropractor emphasized the importance of including mental health discussions when treating patients with complex conditions, although they admitted to discomfort in such conversations:

“*…in some cases where there is a strong psychosocial component to or what appears to be a strong psychosocial component to their problem, (I) try and get into that a little bit. Whether it is….some type of depression or anxiety issues that might be contributing…..I would say as a clinician I am less comfortable talking to patients about psychosocial issues but I have done that with patients from time-to-time especially if that what appears to be a major contributing factor to their problem*” (DC4).

Placing importance on establishing a warm doctor-patient relationship could also explain higher PPOS Caring scores. One chiropractor described how they approach patients of different age groups, using an empathetic approach by imagining how they would want their parents treated in the same scenario:

“*If I see someone who is close to my parent’s age then I just always envision how I would want my mother or father treated when they went to a clinic*” (DC7).

PPOS Sharing subscale scores were lower than the PPOS Caring scores, although chiropractors discussed consideration of patient preferences, demonstrating willingness to adapt to patient needs and values:

“*If I am talking with somebody about lowering their stress level, I am not going to make them go for a walk in the woods if they hate that, we are going to talk maybe about something that is a little bit easier or something that fits in with their life that is going to be an actual valuable tool to them*” (DC5).

“*I always try and tailor the treatment method or treatment approach to the patient and their circumstances*” (DC6).

Table 3 provides comparisons of the mean differences for several variables from the chiropractor questionnaire. There were no significant differences in mean PPOS Overall, Sharing or Caring subscales scores based on gender, chiropractic institution, or style of clinical practice.

**Table 3:** Average PPOS scores for categorical variables

Variable	Average PPOS overall (95% CI)	Average PPOS sharing (95% CI)	Average PPOS caring (95% CI)
Gender^a^			
Female (n = 10)	4.50 (4.10–4.90)	4.29 (3.78–4.79)	4.71 (4.33–5.09)
Male (n = 21)	4.28 (4.10–4.46)	4.16 (3.84–4.47)	4.40 (4.25–4.55)
	t = 1.27 (*p* = 0.216)	t = 0.486 (*p* = 0.631)	t = 2.01 (*p* = 0.054)
Chiropractic institution^a^			
CMCC (n = 25)	4.38 (4.18–4.58)	4.25 (3.96–4.55)	4.51 (4.32–4.70)
Other (n = 6)	4.22 (3.94–4.51)	3.98 (3.34–4.62)	4.46 (4.17–4.75)
	t = 0.76 (*p* = 0.453)	t = 0.865 (*p* = 0.394)	t = 0.248 (*p* = 0.806)
Practice style^a,b^			
Solo (n = 0)	N/A	N/A	N/A
Multi-chiropractor (n = 6)	4.37 (3.88–4.86)	4.30 (3.54–5.05)	4.44 (3.99–4.90)
Multidisciplinary (n = 25)	4.35 (4.16–4.54)	4.18 (3.89–4.46)	4.52 (4.34–4.69)
Other (n = 0)	N/A	N/A	N/A
	t = -0.11 (*p* = 0.91)	t = -0.37 (*p* = 0.71)	t = 0.37 (*p* = 0.72)

PPOS, Patient practitioner orientation scale; CI, Confidence interval; CMCC, Canadian Memorial Chiropractic College

^a^Independent samples t-test, equal variances assumed based on Levine’s test

^b^One-way ANOVA with Scheffe’s S post-hoc test (not used as practice style was collapsed)

*Significant at 0.05

We identified several continuous independent variables with significant correlations of moderate or weak strength with PPOS Overall and subscale scores (Table 4). PPOS Overall, Sharing and Caring subscales scores were all significantly correlated with years in practice. Interview data supported these results, as some chiropractors reported that greater experience was important in developing the patience and skills necessary to see each patient as a unique individual with their specific treatment needs.

*“I think that when you are a little bit more experienced you are a little bit better at managing your time and I also think that you understand a little bit more the importance of spending time with people”* (DC5).

*“….take your time, and acknowledge the patient, that they had a long journey before they came to see you. So, they are going to have to kind of sift through that and be as thoughtful as you possibly can”* (DC4).

**Table 4:** Correlations between continuous variables and PPOS scores (Pearson correlation)

Independent variable	PPOS overall (95% CI)	PPOS sharing (95% CI)	PPOS caring (95% CI)
Years in practice	0.517 (0.199 to 0.736)*	0.457 (0.123 to 0.698)*	0.374 (0.023 to 0.643)
Patients seen per week	0.256 (− 0.108 to 0.56)	0.489 (0.163 to 0.719)*	− 0.246 (− 0.552 to 0.119)
Patients seen per hour	0.333 (− 0.024 to 0.615)	0.528 (0.214 to 0.743)*	− 0.144 (− 0.474 to 0.222)
Weekly hours seeing patients	− 0.167 (− 0.492 to 0.199)	− 0.048 (− 0.4 to 0.312)	− 0.284 (− 0.58 to 0.078

PPOS, Patient practitioner orientation scale; CI, Confidence interval

*Significant at *p* < 0.05

However, other chiropractors described more doctor-centred attitudes and behaviours that developed with more practice experience, for example:

*“I definitely have the anecdotal evidence over the years of 17 years of practice to say this is what has worked over time, this is what I do”* (DC3).

*“Obviously there are lots of pitfalls that we can get into in clinical practice. ….you may or may not have the patience for a certain person, you know, the way they present. So that can harm the care”* (DC1).

*“With experience my approach has differed slightly, the longer that I’ve been in business I find that I almost have to be……I guess just pickier about the exercises and stretches and stuff that I do prescribe and with following up on whether or not that is necessary. I also don’t have an immediate follow-up to see how they are resolving.”* (DC7).

PPOS Sharing scores were also significantly correlated with number of patients seen per week and per hour. This finding may appear to challenge the chiropractors’ interview data that identified concerns that working under time constraints or being too busy could potentially hinder their ability to provide patient-centred care, as noted in the following quotes:

*“…if they (*other chiropractors*) are trying to see a greater number of patients in a certain period of time whether within an hour, a day, or a week, they end up with, there are only so many hours in a day so you end up squishing more patients into a smaller time period, so you spend less time with the patient, and it ends up becoming a lot more like a passive type of approach, conveyor belt type of practice, where if you are only spending 5 min with a patient you can’t educate, coach, reassure, and treat the patient in those visits and you can’t effectively manage a patient with complex chronic problems that way”* (DC6).

“*the busier you are, the busier you make yourself, the less attentive I think you will be to the patient’s needs”* (DC1).

Weekly hours spent seeing patients did not correlate significantly with any of the PPOS Overall or subscale scores.

## Discussion

Our study explored the attitudes and orientation of a small group of practising Canadian chiropractors towards patient-centredness. The mean overall PPOS score among the participating chiropractors was 4.35 (95% CI 4.18–4.52), while the PPOS Sharing score was 4.20 (95% CI 3.95–4.45) and the mean PPOS Caring score was 4.50 (95% CI 4.35–4.66). These average scores fall within the ‘doctor-centred’ range described by Krupat et al. (below 4.57) [28]. Previous studies using the PPOS to assess the attitudes and orientation of medical doctors reported conflicting results, demonstrating higher [28, 35] and lower [30–32] mean overall PPOS scores when compared with our study (see Table 5).

**Table 5:** PPOS scores in studies of medical doctors in comparison with the chiropractors from the current study

First author, year of publication, country	Sample size (n) and profession	PPOS overall score mean (95% CI)	PPOS sharing score mean (95% CI)	PPOS caring score mean (95% CI)
Current study, Canada	31 chiropractors	4.35 (4.18–4.52)	4.20 (3.95–4.45)	4.50 (4.35–4.66)
Wang, 2020, China [30]	617 medical doctors	3.78 (3.74–3.82)	3.09 (3.03–3.15)	4.59 (4.54–4.64)
Wang, 2017, China [31]	71 medical doctors	3.66 (3.52–3.80)	2.94 (2.76–3.12)	4.71 (3.89–5.53)
Krupat, 2000, USA [28]	177 medical doctors	4.80 (4.73–4.87)	4.62 (4.51–4.71)	4.98 (4.91–5.05)
Chan, 2012, Malaysia [35]	12 medical doctors	4.97 (4.92–5.02)	4.65 (4.52–4.78)	5.26 (5.11–5.41)
Abiola, 2014, Nigeria [32]	214 medical doctors	3.98 (3.90–4.06)	4.25 (4.16–4.34)	3.71 (3.63–3.79)
Carlsen, 2006, Norway y[62]	41 medical doctors	N/A	4.31 (4.17–4.45)	N/A

PPOS, Patient practitioner orientation scale; CI, Confidence interval; n, Number in sample

The mean PPOS Sharing score in our study was both higher than previously reported [30, 31] and lower [28, 35] but similar to several other studies involving medical doctors [32, 62] (see Table 5). The mean PPOS Caring score in our study was again similar to those reported in earlier studies of medical doctors [30, 31], while lower [28, 35] and higher [32] than reported in other studies.

Hammerich et al. [37] conducted a survey of 1858 chiropractic students and interns from six countries, finding that mean scores on the PPOS Overall (4.18, 95% CI 4.16–4.20) as well as the Sharing (3.89, 95% CI 3.86–3.92) and Caring (4.48, 95% CI 4.45–4.50) subscales were lower than observed in our study. Mean PPOS Sharing scores from our study were higher than those reported by Hammerich et al. [37], but not mean PPOS Overall or Caring scores. Their study assessed chiropractic trainees and it is unknown whether attitude towards patient-centredness as a student predict future attitudes in practice or if such attitudes change with experience after graduation. Increasing years in practise correlated with higher PPOS scores, suggesting that greater clinical experience could influence practitioner attitudes towards patient-centredness. Patel et al. [63] found that time and practise are necessary for health professionals to become more patient-centred and that learning patient-centred care skills happens primarily on-the job. Our findings support this assertion as we found that years in practice was significantly correlated with chiropractor attitudes and orientation towards patient-centred care in the form of PPOS Overall scores, as well as the PPOS Sharing and Caring subscales. Krupat et al. [28] found that clinical experience was significantly associated with PPOS Overall and Sharing scores as medical doctors in their study with 10 years or less or 21 years or more were more patient-centred than doctors with 11 to 20 years of experience. The reason for the decrease in patient-centred attitudes in years 11 to 20 was uncertain and not observed in our study. However, Krupat et al. [28] did not identify significant differences between the different years of experience (younger and older clinicians) and PPOS Caring scores.

The weak but significant correlation between PPOS Caring scores and years in practice in our study was similar to previous findings among Nigerian medical doctors [32]. Practitioner experience could be important in developing more patient-centred attitudes towards a holistic approach to care and chiropractor-patient relationships. Our interview data reflected differing viewpoints from chiropractors on how their experience influenced their attitudes and behaviours in practice, and this may explain why only a weak correlation was observed. Some chiropractors felt time in practice spent with patients was necessary to develop more patient-centred attitudes, while others described more doctor-centred attitudes and potentially doctor-centred habits that evolved over time. Younger practitioners may be adept at many aspects of patient care but may still be learning and refining their communication skills and ability to efficiently manage information.

We identified that PPOS Sharing scores from chiropractors in our study were significantly correlated with seeing more patients per hour and per week. PPOS Caring and Overall scores were not significantly correlated with the weekly or hourly patient loads. The significant associations with higher weekly or hourly caseloads and PPOS Sharing scores contradicted the expectations described by chiropractors during interviews that seeing higher patient loads might reflect less patient-centred attitudes and result in lower quality care. Some chiropractors expressed concern that high patient volumes may compromise the ability of their colleagues to deliver patient-centred care. A systematic review by O’Malley and colleagues [64] identified practitioner management of capacity and workload among several provider-level characteristics associated with exceptional care when assessed using the Positive Deviance approach.

The apparent dissonance between seeing greater patient volumes and higher PPOS Sharing scores may reflect the ability of experienced chiropractors to efficiently deliver patient-centred care, even within time constraints. Schafer et al. [65] conducted a survey of general practitioners’ practices in 33 countries (n = 67,873 patients) identified positive associations between general practitioners who work more hours per week and patient perceptions of better communication and receiving comprehensive care, and indicated that this may be reflect that general practitioners who keep more weekly clinical hours may have better availability and a better understanding of their patients and their health. It is similarly possible that, while seeing more patients, chiropractors have adapted their approach to ensure that key patient-centred elements such as shared decision-making are maintained. It is also possible that while chiropractors may strive to maintain patient-centred practices, the practical realities of high patient loads could limit the time and attention given to each patient, which may be reflected by the lack of significant correlation with PPOS Caring scores. As such, it is uncertain whether chiropractors who are busier and have more patient-centred attitudes toward patient involvement in decision-making and sharing information provide care to patients that reflects those attitudes. This is an important area for further research to determine the manners in which chiropractors attempt to balance patient loads with maintaining care quality.

We report here that the chiropractors’ mean PPOS scores were not significantly different between female and male on the PPOS Overall, Sharing, and Caring scores. Interview data from one male chiropractor reflected a belief that female chiropractors may be more patient-centred. Female health professionals tend to be more empathetic and patient-centred than males [66–68], with previous research demonstrating significantly higher PPOS scores among female medical doctors [28, 43] and chiropractic trainees [37]. However, as in our study, other investigators have found apparently higher, albeit non-significant differences in PPOS Overall and subscale scores favouring female medical doctors compared with male doctors [30–32, 62]; with one exception that found male doctors in China had non-significantly higher PPOS Caring scores [31]. Thus, to date there is no consensus as to whether the gender of health professionals has a significant impact on PPOS scores.

The interview data provided valuable insights into how chiropractors integrate patient-centred care in practice. A key concept that emerged from the interviews was the role of patient autonomy and shared decision-making. Chiropractors often described their efforts to tailor care based on individual patient preferences and interests, ensuring that treatment aligns with the patient’s values and comfort level.

Chiropractors employing an holistic care approach also emerged as a prominent concept. Chiropractors described how they consider not only a patient’s physical symptoms and conditions but as much of their life as they can. This holistic approach aligns with the biopsychosocial model of care, which emphasizes the importance of considering and addressing the mental/psychological and social dimensions of health in addition to the physical condition. When psychosocial factors are recognized as playing a significant role, chiropractors mentioned exploring mental health and lifestyle factors.

As the PPOS reflects the attitudes of respondents towards patient-centred care [Krupat], it would be reasonable to question whether attitudes are reflective of the actual provision of patient-centred care. A validation study conducted by Shaw, Woiszwillo, and Krupat [50] asked a small number of HCPs (n = 14 including 1 chiropractor) and back pain patients to complete the PPOS and then recorded verbal exchanges between those clinicians and patients and found that HCPs with more patient-centred attitudes as demonstrated on the PPOS (i.e., higher PPOS scores) asked significantly more questions related to lifestyle/psychosocial issues of patients and tried to establish rapport more frequently with patients, and asked significantly fewer biomedical questions than HCPs with lower PPOS scores.

Our findings could have been impacted by potential confounders that we did not consider. Specifically, patient demographic, cultural, workplace, geographic, or socioeconomic variables were not assessed in our questionnaire, and may have an influence on chiropractor PPOS scores. Chiropractors may adjust their communication and care strategies based on the patient’s background. As an example, one chiropractor described how they approach patients of different age groups, using an empathetic approach by imagining how they would want their parents treated in the same scenario.

Patient occupation provided an example of chiropractors considering individual patient circumstances as they attempt to ensure that recommendations are relevant and applicable to the patient’s daily life. Chiropractors frequently discussed the role of a patient’s occupation, work environment, and physical demands in shaping their health conditions, the doctor-patient relationship, and possible interventions. Although the impact of other patient demographic, culture and gender or socioeconomic background factors were not explicitly explored, individual patient preferences, which could stem from needs arising due to those factors, play a potential role in the chiropractor’s patient-centered approach. Chiropractors emphasized that care must be individualized, respecting patient choices and comfort, and this could relate to those variables.

### Strengths and limitations

An important limitation of our study is the small sample size (n = 31) which may have resulted in a lack of power to detect a true difference or association if one exists. Therefore, our findings should be interpreted with caution due to the high risk of a Type II error, particularly for non-significant results. We used a small convenient sample of chiropractors, and thus the results may not be generalizable to all chiropractors. However, one of the strengths of this study was the representativeness of the sample of Canadian chiropractors. Our survey sample was similar to previous surveys of Canadian chiropractors in gender distribution, place where they received their chiropractic training, average years in practice and patients seen per week, and average weekly hours spent seeing patients [69, 71, 72]. One noticeable difference between our sample and a previous Canadian national survey of chiropractors [69] was in the types of practices where the chiropractors work. Over 80% of the respondents in our study practiced in multidisciplinary settings, and the rest in multi-chiropractor clinics, with none in solo practice or other settings. The national survey data [69] indicated that half of Canadian chiropractors work in multidisciplinary clinics, while one third were in solo practice, and the rest in multi-chiropractor clinics. Based on our data, we estimated a minimum sample size using Cochran’s sample size formula [73] for continuous survey data, calculating an estimated minimum sample size of 323 for a future survey of Canadian chiropractors.

Among the chiropractors who participated in the interviews, most were male and graduated from Canadian chiropractic educational institutions, which could affect the generalizability of the interview findings to other settings and populations. In addition, we were unable to determine the response rate for this study as we did not ascertain the number of chiropractors working at each participating clinic. It is possible that non-respondents may have differed from respondents in their attitudes as measured with the PPOS.

Several of the chiropractors involved in the study were known to the lead author, which may have led to concerns about sampling bias and generalisability to other chiropractors in the field. Furthermore, interviewed chiropractors were known to the interviewer and aware that the interviewer was a chiropractor, which may have influenced their responses and created researcher bias and/or social desirability response bias [74]. We did not consider PPOS scores when selecting the chiropractors we interviewed, including participants with a wide range of PPOS scores may have provided more diverse perspectives on person-centered care. To minimize potential response bias, participants were assured of the anonymity of their survey responses, and efforts were made to create an environment in which they could share their experiences openly. Furthermore, reflexivity and journalling were employed by the primary investigator to mitigate the influence of pre-existing professional relationships on the data interpretation. We also did not employ member checking of the transcripts nor analysis, which could affect the credibility of the qualitative data [75]. However, we established credibility in our qualitative data collection and analysis through bracketing and reflexivity [55]. Furthermore, we used investigator triangulation during qualitative data analysis as two authors, one of whom was not a chiropractor, independently coded and analyzed the data. We ensured validity of the qualitative data through constant comparison [76].

Finally, Krupat et al. [28], do not provide a detailed rationale for their scoring bounds for the “doctor-centred”, “patient-centred”, and “medium” categories despite being so cited in most studies using the PPOS. Given these potentially subjective scoring bounds future research should attempt to further clarify whether use of these categories is appropriate.

## Conclusions

This is the first study to use the PPOS to measure chiropractors’ patient-centred attitudes and approach to care. Chiropractors’ scores of attitudes towards patient-centredness were comparable to those reported in other health professions. Our findings suggest that more years of practice experience and increased hourly and weekly patient load were associated with more patient-centred attitudes, particularly for PPOS Sharing scores, indicating that attitudes towards patient involvement in decision making may be learned through greater clinical experience with patients. Interview data largely corroborated the quantitative findings.

This study is exploratory in nature and should be viewed as a first step in understanding chiropractor attitudes toward PCC. The small sample and convenient sample limit the generalizability of the results, and future research with larger, more representative sample is needed. Future research should explore chiropractor attitudes and orientation towards care using the PPOS or a similar instrument on larger samples sizes with consideration of additional confounding factors. A future study where both chiropractors and some of their patients complete the PPOS would also be beneficial in determining the alignment between patient and practitioner attitudes toward PCC. Further explorations of chiropractors’ attitudes and orientation towards care could inform the education and development of trainee and registered chiropractors and increase the potential for more PCC. Our research supports existing evidence on the need for additional educational opportunities in PCC for trainees and practising clinicians [36, 77]. Interventions that encourage providers to offer more PCC can improve the quality of care provided, improve their job satisfaction, and reduce HCP stress and burnout and warrant further research and implementation [78]. Thus, interventions that enhance student and provider knowledge and skill acquisition in PCC should continue to be considered in curriculum design, implementation, and evaluation [78]. Further assessing whether more patient-centred attitudes towards care from practitioners are associated with patients receiving care that is more patient-centred should also be considered.

## Supplementary Information

Below is the link to the electronic supplementary material.

Supplementary Material 1

Supplementary Material 2

## Data Availability

The datasets used and analyzed during the current study are available from the corresponding author on reasonable request.

## Abbreviations

PCC: Patient-centred care
HCP: Health care practitioner
PPOS: Patient-practitioner orientation scale
SD: Standard deviation
CI: Confidence intervals
