# Supplementary material for: An explanatory mixed methods study assessing Canadian chiropractors’ attitudes and orientation toward patient-centred care
Source: Chiropr Man Therap. 2025 Oct 22;33:48. doi: 10.1186/s12998-025-00610-2 (PMC12542363; doi:10.1186/s12998-025-00610-2)
Supplement: Supplementary file 1 — Supplementary Material 1 [file 12998_2025_610_MOESM1_ESM.docx]

Appendix 1. Individual chiropractor interview guide

Chiropractor Individual Interviews

**Introduction (5-10 minutes):**

Discuss and collect signed informed consent prior to audio recording – confirm that individuals will not be identified, none of the quotes attributed to individuals personally. Ask if participant has any questions about the reason for, or content of, the informed consent. Subjects will need to sign the informed consent form prior to commencement of the interviews**.**

**Introductory Script:**

**Welcome**

“Good morning/afternoon and welcome. Thanks for taking the time to join me to talk about chronic musculoskeletal conditions and chiropractic care.

**Purpose**

The purpose of this interview is to obtain your perspectives and experiences in working with patients with chronic musculoskeletal conditions and how patients are involved in the decisions that are made surrounding their care. We also want to find out about the care you provide for these patients and how you view their role in such care. Last year you completed a questionnaire that asked you about this, and now we’re here to discuss it and learn about in your own words.

**Guidelines**

We're audio-recording the session because I don't want to miss any of your comments. People say really helpful things in these interviews, and I can't write fast enough to get them all down. To maintain confidentiality and privacy, I ask that you do not disclose where it is that you were recruited from or the name of the clinic or city where you work.

I’ve got a number of questions that I want to ask, but my job is really to listen. This will be more interesting if we treat this like a conversation. I may need to interrupt to get through all the questions. I apologize ahead of time if I need to do this.

There are no right or wrong answers, only different points of view. Keep in mind that we're just as interested in negative comments as positive comments, and at times the negative comments are the most helpful.

Please be aware during this discussion, that anything declared which is illegal or indicates unprofessional practice will need to be passed on to the appropriate authorities for action.

Alright let’s begin…I will start with an easy question…[ice breaker question]”

**Core Interview Questions (40-50 minutes):**

1. What is a typical visit like when seeing a patient with a chronic musculoskeletal condition?

- What types of things do you talk about?
  - Do you ask patients about how their chronic condition affects their life? (Problem-Solving/Contextual)
  - Do you discuss their health habits? (Goal setting)

1. In what ways are patients with chronic musculoskeletal conditions different from those with an acute condition?
   - - Does your approach differ between these patient populations?
     - Do you have an example that might illustrate this?
     - What are your expectations when caring for a patient with chronic pain?
2. How do you design treatment plans for patients with chronic msk conditions?
   - What role do patient values play in the plan?
   - Are a patients’ personal, social and/or occupational circumstances considered when making recommendations? Describe. (Problem-Solving/Contextual)
   - What role do patients play in decision-making about their care? (Patient Activation)
     - - Do you ask for or take patient ideas into consideration when coming up with a treatment plan? (Patient Activation)
3. How do you provide care that goes beyond what happens in the clinic? (Follow-up/Coordination)
   - How does your treatment plan enable patients to implement during their daily life and between visits? (Problem-Solving/Contextual)
     - - What take-home resources or advice do you give your patients?
       - Do you give your patients information to enhance their understanding of their condition and the care for it? (Delivery system design/Decision support)
       - Do you give information to patients? Describe (probe: such as a written list of exercises or other things to improve patient health, even websites to consult? (Delivery system/decision support)
       - Do you recommend specific groups or classes in the community that may help with their condition? (yoga, pilates, tai chi, support groups) (Follow-up / coordination)
   - What role does following-up with patients play in their care? (Follow-up / coordination)
     - - What is your process? Why do you follow-up in this manner?
       - How often do you follow-up?
4. Do you and your patient establish goals in caring for their condition? (Goal setting)

- What kinds of goals do you set?
- Do you write those goals down as part of a treatment plan and monitor them? (Goal setting)

1. Do you communicate and coordinate care with other health care professionals for patients with chronic msk conditions? Describe (Follow-up / coordination)

- Could you give an example of when you have done this?
- Is there a role for the patient to play in the collaboration between care-givers? How?

1. Are there any characteristics of chiropractors that you think could influence how attentive they are to a patient’s specific needs? (Patient-as-person)
   - E.g. how busy they are, how long they’ve been in practice, their gender, the office environment and practice atmosphere
   - Do you think the length of time that you’ve been seeing a patient influences how they view working with you on their health concerns? (Therapeutic Alliance)
     - - Could you tell me more about that?
2. What is your opinion about how patient satisfaction is affected by how well they think that you understand them and their specific health care needs? (Patient-as-person)
   - - Please describe why you think this might be
     - Do you think that seeing a patient with a chronic condition more often would influence your doctor-patient relationship? (sharing power and responsibility)
     - Could you explain that further?
3. If you had one minute to give advice to other chiropractors working with patients with chronic musculoskeletal conditions, what would you say?

- Why do you say that?

**Conclusion (5-10 minutes)**

**Summary (3 minutes or less)**

- Here is a brief summary of the main points we heard today…..[insert here]…..Is there anything I have missed? Does that reflect the conversation we had? What would you add to or change about that summary?
- Do you have any final comments regarding your experiences treating patients with chronic musculoskeletal conditions that you would like to add?
- Do you have any recommendations to other chiropractors to help them become more patient-centered?
- Verbal acknowledgement and thank you:

“We have come to the end of our interview. Once again I’d like to thank you for joining me today and sharing your experiences so openly and honestly.
