# Supplementary material for: An explanatory mixed methods study assessing Canadian chiropractors’ attitudes and orientation toward patient-centred care
Source: Chiropr Man Therap. 2025 Oct 22;33:48. doi: 10.1186/s12998-025-00610-2 (PMC12542363; doi:10.1186/s12998-025-00610-2)
Supplement: Supplementary file 2 — Supplementary Material 2 [file 12998_2025_610_MOESM2_ESM.docx]

Appendix 2. *Good Reporting of a Mixed Methods Study (GRAMMS) guide* [56]*.*

| **Guideline** | **Section** |
| --- | --- |
| Describe the justification for using a mixed methods approach to the research question | Methods |
| Describe the design in terms of the purpose, priority and sequence of methods | Methods |
| Describe each method in terms of sampling, data collection and analysis | Methods – Quantitative component and Qualitative component sections |
| Describe where integration has occurred, how it has occurred and who has participated in it | Methods – Reporting and integration section |
| Describe any limitation of one method associated with the presence of the other method | Discussion - Limitations |
| Describe any insights gained from mixing or integrating methods | Discussion |
